# A Comprehensive Evaluation Algorithm of Multi-Point Relay Based on Link-State Awareness for UANETs

**DOI:** 10.3390/s24051702

**Published:** 2024-03-06

**Authors:** Rencheng Jin, Xinyuan Zhang, Jiajun Liu, Guangxu Wang, Di Zhang

**Affiliations:** Key Laboratory for Micro/Nano Technology and System of Liaoning Province, Dalian University of Technology, Dalian 116024, China; xyzhang_4869@mail.edu.cn (X.Z.); liujiajun@mail.dlut.edu.cn (J.L.); wgx13831057446@mail.dlut.edu.cn (G.W.); zhangdi_tyut@163.com (D.Z.)

**Keywords:** UANETs, MPR, OLSR, mobility, link status, NS-3, TOPSIS

## Abstract

The Multi-Point Relay (MPR) is one of the core technologies for Optimizing Link State Routing (OLSR) protocols, offering significant advantages in reducing network overhead, enhancing throughput, maintaining network scalability, and adaptability. However, due to the restriction that only MPR nodes can forward control messages in the network, the current evaluation criteria for selecting MPR nodes are relatively limited, making it challenging to flexibly choose MPR nodes based on current link states in dynamic networks. Therefore, the selection of MPR nodes is crucial in dynamic networks. To address issues such as unstable links, poor transmission accuracy, and lack of real-time performance caused by mobility in dynamic networks, we propose a comprehensive evaluation algorithm of MPR based on link-state awareness. This algorithm defines five state evaluation parameters from the perspectives of node mobility and load. Subsequently, we use the entropy weight method to determine weight coefficients and employing the method of Technique for Order Preference by Similarity to Ideal Solution (TOPSIS) for comprehensive evaluation to select MPR nodes. Finally, the Comprehensive Evaluation based on Link-state awareness of OLSR (CEL-OLSR) protocol is proposed, and simulated experiments are conducted using NS-3. The results indicate that, compared to PM-OLSR, ML-OLSR, LD-OLSR, and OLSR, CEL-OLSR significantly improves network performance in terms of packet delivery rate, average end-to-end delay, network throughput, and control overhead.

## 1. Introduction

In recent years, propelled by the rapid advancements in drone technology, Unmanned Aerial Vehicles (UAVs) have witnessed widespread adoption across various sectors, including aerial photography, 5G communication, agricultural and forestry monitoring, as well as search and rescue operations [1,2]. In order to efficiently complete tasks, multiple drones need to establish Unmanned Aerial Vehicle Ad hoc Networks (UANETs) in the actual work process to support real-time and efficient collaborative communication between each other [3]. UANETs, a variant of the well-established Mobile Ad hoc Networks (MANETs), represent a prominent trend in wireless communication due to their diverse range of applications [4]. Compared to traditional mobile ad hoc networks, UANETs exhibit stronger mobility and are not restricted by terrestrial factors when moving in the air, leading to more frequent changes in network topology [5]. In UANET, the routing technology at the network layer is one of the core technologies. However, existing routing protocols are mostly designed for static networks and do not require real-time updates of link states [6], rendering them inadequate for the swift mobility characteristic of UANETs. Therefore, it is necessary to optimize and improve the existing routing protocols based on the current network operation status.

OLSR is a proactive, table-driven multi-hop routing protocol [7]. In the network, information is mainly exchanged in the form of HELLO messages and Topology Control (TC) messages [8]. The process of link detection and neighbor discovery between nodes is accomplished through broadcasting HELLO messages. The MPR nodes forward TC messages to obtain link information in the network, ultimately establishing and maintaining the entire network topology, and applying relevant path algorithms to generate routes [9]. The MPR mechanism is the core technology of the OLSR protocol, effectively reducing message flooding [10]. This protocol is suitable for network applications requiring short-term concurrent transmission and low latency, and it is applicable to large-scale networks with high node density.

In static sensor networks, research on topology control is relatively extensive. However, in dynamic networks, the transient nature introduced by node mobility leads to frequent changes in network topology, resulting in significant impact on network topology and posing great challenges to topology control [11]. Meanwhile, due to the rapid node mobility and frequent network topology changes in UANETs, direct use of the OLSR protocol often leads to increased likelihood of link failures, high network topology changes, and delays [12]. Moreover, only nodes selected as MPR, known as a message sorting station in the network, can forward control messages such as TC messages, while other ordinary nodes cannot [13]. At the same time, MPR nodes can generate link state information between themselves and MPR selection nodes. Therefore, research on MPR is highly necessary as it directly affects network performance.

The structure of the remaining sections in this paper is as follows: Section 2 provides a review and analysis of the current related work. Section 3 introduces the relevant evaluation parameters and the process of the MPR selection algorithm in detail. In Section 4, the simulation process is elaborated, followed by a discussion and analysis of the simulation results. Finally, Section 5 summarizes the paper and identifies future research trends.

## 2. Related Works

### 2.1. Research Status

5G communication is the latest generation of cellular mobile communication technology, and its greatest value lies in driving the digital transformation of various industries, enabling a shift from personal mobile applications to industry applications [14]. At present, some research work on combining 5G communication and wireless communication networks is as follows.

Reference [15] explores Vehicle Ad hoc Networks (VANETs) within 5G systems, presenting a dynamic vehicle resource allocation algorithm that considers the dynamic mobility characteristics of vehicular nodes. This approach enhances the practicality and scalability of the network while ensuring a rational distribution of network resources. Reference [16] delves into cooperative platooning scenarios within VANETs integrated with 5G communication. It proposes a power control algorithm based on distributed dynamic programming, taking into account the mobility of vehicle nodes, to achieve fair resource allocation within base stations and each platoon. Considering the impact of node mobility. Reference [17] investigates session continuity in 5G communication systems under scenarios involving dense and mobile networks. The findings highlight the significant effect of communication session continuity, attributed to link interruptions caused by node mobility and frequent blocking due to data retransmissions. The advancement of 5G technology provides high-speed, low-latency, and reliable communication support for drone networks.

In recent years, some researchers have considered improving the MPR mechanism from various aspects when studying the OLSR protocol, including selecting appropriate metric parameters, altering selection strategies, and the impact of link variations. The current research status is as follows.

References [18,19,20] explore the impact of node mobility on the MPR mechanism and propose novel metric parameters for optimization. Reference [18] introduces the LD-OLSR protocol, leveraging link duration and three-dimensional node situational data to forecast link durations. By incorporating node forwarding willingness, it introduces an MPR factor, effectively enhancing packet delivery rates and reducing latency. Reference [19] presents an efficient MPR selection algorithm considering node mobility’s effect on network topology. It introduces the concept of “effective coverage area”, estimating future node positions using historical data to expedite network topology establishment and reduce TC message redundancy. Reference [20] designs the mobility and queue-length-aware MP-OLSR protocol based on multi-criteria decision-making metrics, considering various influencing factors.

References [21,22,23] enhance the MPR mechanism by altering the selection strategy. Reference [21] proposes a reverse-thinking MPR selection algorithm, combining iterative and set operations to eliminate redundant nodes effectively, enhancing data transmission success rates. Reference [22] introduces a novel MPR node selection method employing Self-Organizing Map (SOM) artificial neural networks to distinguish strong and weak MPR nodes and select reliable retransmission-capable nodes, improving throughput, packet delivery rates, and network security. Reference [23] presents the Dynamic Updating Ant Colony Optimization (DUACO) algorithm incorporating state information and dynamic update mechanisms, mitigating MPR set redundancy and enhancing network performance.

References [24,25,26] enhance the MPR mechanism considering link variations. Reference [24] proposes a link stability-based MPR selection algorithm, prioritizing nodes with stable link quality to extend the MPR set’s effective time and reduce topology change impacts on data transmission. Reference [25] introduces the Multi-dimensional Perception and Energy-Aware OLSR (MPEAOLSR) routing protocol, addressing network challenges like frequent topology changes and congestion by considering link conditions and energy awareness. Reference [26] proposes an M-OLSR routing protocol based on the SL-MPR selection algorithm, considering node mobility and link variations to tackle issues in current UANETs, such as topology changes and control message redundancy.

While many scholars have addressed the selection of MPR nodes considering factors like link status, network load, and node energy conditions, utilizing neural network optimization algorithms and leveraging multiple parameters for decision-making, there remains a gap in proposing a comprehensive evaluation algorithm grounded in multi-dimensional perception. Such an algorithm would integrate various indicator parameters from different aspects of the same evaluation object to derive a holistic evaluation metric.

Therefore, it is imperative to propose a multi-attribute comprehensive evaluation routing algorithm based on multi-dimensional state perception for UANETs operating in highly dynamic and rapidly changing topology scenarios.

### 2.2. MPR Selection Model

**Figure 1:**
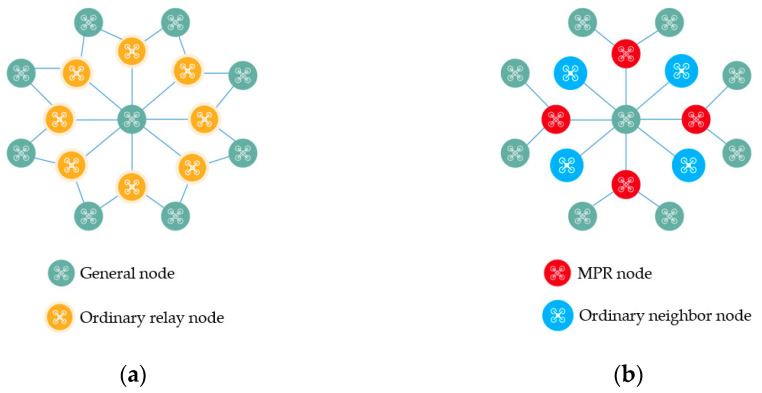
The comparison of data transmission links under the flooding mechanism and MPR mechanism. (**a**) Diagram of data transmission link under the flooding mechanism; (**b**) schematic diagram of data transmission link under MPR mechanism.

In OLSR, nodes are categorized into MPR nodes and general nodes based on their ability to forward control messages. General nodes are limited to receiving and processing messages, while only nodes designated as MPRs have the capability to forward control messages [27]. Therefore, selecting appropriate MPR nodes is pivotal for enhancing network performance. The MPR mechanism effectively limits the widespread dissemination of control messages, thereby reducing control overhead in the network, preventing resource wastage, and mitigating network congestion [28]. As illustrated in Figure 1, employing the MPR mechanism for flooding substantially decreases the transmission of control packets while covering all nodes. Furthermore, as the network expands, the benefits of this mechanism become even more pronounced.

$N_{1}(i)$ means 1-hop neighbor set of node i, $N_{2}(i)$ means 2-hop neighbor set of node i, and M(i) means MPR set of node i. The steps for selecting MPR nodes are as follows [29]:

**Step 1.** Select nodes from $N_{1}(i)$ through which node i can only reach certain 2-hop neighbors, and then add them to M(i).
**Step 2.** Sort 1-hop neighbors from high to low based on the number of the coverage for 2-hop neighbors, and select the ones with the highest coverage to join M(i).
**Step 3.** Update and remove the 1-hop neighbors from $N_{1}(i)$ and 2-hop neighbors from $N_{2}(i)$ for each addition operation.
**Step 4.** Repeat the Step 2 and remove nodes through the Step 3, and finally end operation until the nodes of M(i) can completely cover all of the 2-hop neighbors from $N_{2}(i)$.

The ultimate objective of MPR selection within the 1-hop neighbor set of a node is to ensure the connectivity of data transmission links by achieving full coverage of the 2-hop neighbor set of the node in the network [30].

**Figure 2:**
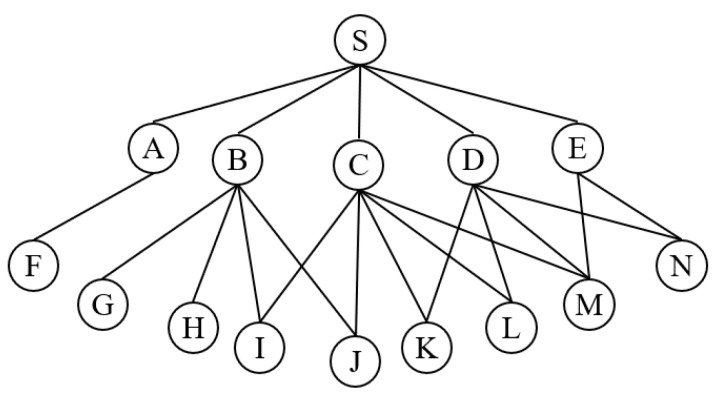
The traditional process of selecting MPR set for node S.

As depicted in Figure 2, the MPRs for node S are chosen from its 1-hop neighbor set, where nodes A to E represent 1-hop neighbor nodes, and nodes F to N represent 2-hop neighbor nodes. Initially, node A, the sole neighbor capable of reaching 2-hop neighbor F, is selected to join the MPR set. Subsequently, nodes are arranged based on their coverage, with nodes C, B, and D sequentially added to the MPR set. Ultimately, the MPR set of node S comprises {A, C, B, D}. It is worth noting that some nodes might receive the same control message forwarded by different nodes, and traditional selection algorithms may not always yield the optimal and minimal MPR set. In the scenario described above, the set {A, B, D} could also achieve full coverage of the 2-hop neighbors.

### 2.3. Analysis of MPR Selection Issues

The measurement for selecting MPR nodes is single, and even when considering multiple factors, the probability of all factors being good is very low. Therefore, it is necessary to integrate multiple factors.

**Figure 3:**
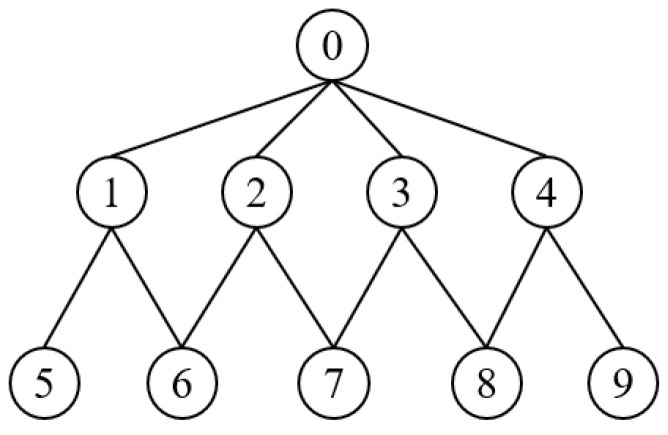
The process of selecting MPR set for node 0.

In the process of selecting MPR nodes, there are nodes with the same initial coverage and current coverage. In this case, a node is randomly selected as the MPR node. If MPRs are selected based on coverage, as shown in Figure 3, there will be two results: {1, 2, 4} and {1, 3, 4}, from which a selection will be made randomly. When the mobility of node 3 is too fast, it is not within the communication range of node 0 at a certain moment, causing link breakage between them. Meanwhile, compared with node 3, if the link duration of node 2 is longer and the link stability is better, then the former reflects a better effect. If a large amount of packets are received at a certain moment resulting in the buffer queue to be full, node 2 can be unable to continue receiving and only discard the packets arriving at the next moment, which will cause packets loss. Considering the load situation of node 2, the latter has a better effect.

Relying solely on single indicators such as node coverage, node mobility, and node load to select MPR nodes is unreasonable [31]. The above examples illustrate that if multiple factors are not comprehensively considered, it will not only affect the selection of MPR nodes, but also disrupt normal communication and data transmission in the network, leading to poor network robustness and decreased overall network performance.

## 3. Proposed Algorithm

Due to the transient characteristics introduced by node mobility in dynamic networks, the network topology undergoes frequent changes. Existing research on dynamic network routing algorithms has paid limited attention to node mobility and network load variations. Additionally, there are a lack of studies that select multiple parameter indicators and utilize multi-attribute decision-making to optimize the MPR mechanism. Therefore, addressing the aforementioned issues, this paper combines the TOPSIS method to establish a multi-attribute comprehensive evaluation model. Leveraging link-state awareness among nodes, a novel MPR selection algorithm named TOPSIS-MPR is proposed. This algorithm emphasizes two key dimensions of link awareness: mobility and load, enabling real-time monitoring of the availability, quality, and data payload of each network link. By integrating multiple parameters and multi-attribute decision-making methods, this research further optimizes the MPR mechanism for dynamic networks.

The formulas and conclusions derived in the paper are based on the following three hypotheses, namely:

**Hypothesis 1.**  *Each drone node is equipped with a Global Positioning System (GPS) that can sense motion and analyze link status based on the location and velocity information provided by the module.*

**Hypothesis 2.**  *The effective communication distance of each drone node is the same, and the signal propagation follows a free space propagation loss model. The received signal power is mainly related to the distance between them.*

**Hypothesis 3.**  *There are many data frames in the MAC layer buffer of each drone node, mainly including data frames waiting to be sent, data frames waiting to be forwarded, control frames waiting to be sent, retransmitted data frames, and confirmation frames waiting to be sent. The length of the queue buffer of a node can reflect its load situation.*

### 3.1. Evaluation Parameters

#### 3.1.1. Awareness of Mobility

Considering the impact of node mobility in the network from the dimensions of time and space, we propose three measurement parameters: link duration, stability degree of link, and average neighbor set change rate.

1. Link Duration

Given the propensity for drone nodes to move at high speeds, they frequently venture beyond the communication range of a node, leading to link disconnections. In light of this, we introduce the concept of *link duration* (LD), defined as the period from the establishment of a connection between two nodes in the network until the disconnection of the link. This metric serves to quantify the stability and reliability of network connections in dynamic environments.

**Figure 4:**
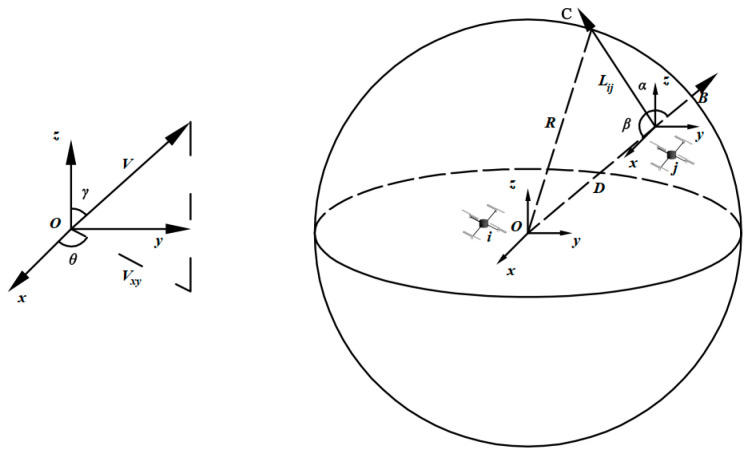
The schematic diagram of relative motion between drone node i and node j.

As shown in Figure 4, we establish a three-dimensional Cartesian coordinate system with node i as the reference center, where node j moves around node i. At time $t_{1}$, node j establishes a link connection with node i at point B, and then moves along the $\vec{V}$ direction to point C at time $t_{2}$. At the next moment, it will disconnect from node i, which will have exceeded the communication range of node j.

Assuming that the measured coordinates of the drone node i and node j are $\left(x_{i},y_{i},z_{i}\right)$ and $\left(x_{j},y_{j},z_{j}\right)$, and the velocities are $\left(v_{ix},v_{iy},v_{iz}\right)$ and $\left(v_{jx},v_{jy},v_{jz}\right)$, respectively, the relative position vector $\vec{OB}$ between node i and node j is denoted as $\left(d_{x},d_{y},d_{z}\right)$, and the relative velocity vector $\vec{OC}$ between node i and node j is denoted as $\left(v_{x},v_{y},v_{z}\right)$.

The expressions are as follows, where α is the angle between the relative position vector $\vec{OB}$ and the relative velocity vector $\vec{OC}$, and β is its complementary angle:

(1) $$\cos\alpha =\frac{\vec{OB}\cdot \vec{V}}{\left|\vec{OB}|\cdot |\vec{V}\right|}$$

(2) $$\cos\beta =-\cos\alpha =-\frac{\vec{OB}\cdot \vec{V}}{\left|\vec{OB}|\cdot |\vec{V}\right|}$$

The LD between node i and node j called $LD_{ij}$ is expressed as:

(3) $$LD_{ij}=\frac{-(v_{x}d_{x}+v_{y}d_{y}+v_{z}d_{z})+\sqrt{\left(v_{x}^{2}+v_{y}^{2}+v_{z}^{2})R^{2}-[{\left(v_{x}d_{y}-d_{x}v_{y}\right)}^{2}+{\left(v_{x}d_{z}-d_{x}v_{z}\right)}^{2}+{\left(v_{y}d_{z}-d_{y}v_{z}\right)}^{2}\right]}}{v_{x}^{2}+v_{y}^{2}+v_{z}^{2}}$$

When two connected nodes maintain consistency in their movement direction and speed, the link maintenance time is longer, making the link less likely to disconnect. Conversely, the link is more prone to disconnection if there is inconsistency in their movement. Therefore, selecting neighbors with larger LD values results in longer link connection times along their paths, ensuring more stable and reliable data transmission.

2. Stability Degree of Link

Link duration assesses link stability by gauging the current or anticipated motion state of the link. However, to further enhance the evaluation of link stability, we propose the *stability degree of link* (SDL), which quantifies the fluctuation level of relative node positions. By leveraging SDL, we can enhance packet delivery rates, diminish data retransmission instances, elevate routing success rates, and ultimately enhance network performance.

Before defining link stability, we first provide the definition of distance variation based on the Chebyshev inequality in statistical theory, which can reflect the fluctuation of distance between node i and node j. The definition of $\delta d_{ij}$ is as follows:

(4) $$\delta d_{ij}=\frac{1}{n-1}(\sum_{t-T}^{t}d_{ij}^{2}-n{\bar{d_{ij}}}^{2})$$

In the above equation, $d_{ij}$ and $\bar{d_{ij}}$, respectively, on behalf of the distance and average distance between node i and node j during time period [t−T,t], and n is the number of distance measurements at the same time period.

To characterize the impact of distance variation on link communication quality, we introduce a distance step function $\varphi (\bar{d})$. This function signifies that as nodes draw closer, the communication quality of the link improves, with distance variation exerting a more pronounced impact on link communication quality.

(5) $$\varphi (\bar{d})=\left\{\begin{array}{c} 1.5,d_{min}\leq \bar{d}\leq \frac{1}{2}R\\ 1.2,\frac{1}{2}R<\bar{d}\leq \frac{3}{4}R\\ 1,\frac{3}{4}R<\bar{d}\leq R \end{array}\right.$$

After introducing the step function $\varphi (\bar{d})$, the definition of link stability between node i and node j called $SDL_{ij}(t)$ is as follows:

(6) $$SDL_{ij}(t)=\frac{\varphi (\bar{d})}{\delta d_{ij}}$$

3. Average Neighbor Set Change Rate

The rapid movement of drone nodes can result in alterations in network topology, frequent shifts in neighboring nodes, and an increased probability of link disruptions, thereby diminishing network performance.

For this reason, we propose the concept of *average neighbor set change rate* (ANSCR) by monitoring the changes in the neighbor set of nodes over a period of time, which can measure the topological changes of its surrounding neighbors. It refers to the change rate of the neighbor set per unit time, expressed as follows:

(7) $$ANSCR_{i}=\frac{INC_{i}+DEC_{i}}{N_{i}(t_{2}-t_{1})}$$

Among the above, $INC_{i}$ represents the number of newly added nodes in the neighbor set of node i during time period $\left[t_{1},t_{2}\right]$, while $DEC_{i}$ represents the number of decreased nodes at the similar period of time. $N_{i}$ represents the number of nodes in the neighbor set of node i at time $t_{2}$. All of these can be obtained by monitoring the neighbor table content of node i.

To reflect time correlation and prevent drastic changes caused by sudden changes in the values of $INC_{i}$ and $DEC_{i}$, we use an exponential moving average strategy for $ANSCR_{i}$. The final expression is as follows, where ξ is the smoothing factor:

(8) $$ANSCR_{i}(t)=\xi \cdot ANSCR_{i}(t)+(1-\xi )\cdot ANSCR_{i}(t-1)$$

The above expression means that the smaller the value of the ANSCR, the smaller the change in neighbors around the node, and the lower the degree of topology change.

#### 3.1.2. Awareness of Load

As the number of data packets sent by nodes in the network increases, the receiving end nodes are unable to process them in a timely manner, making for an increase in the workload of the sending nodes. The escalation in data packet transmission within the network inherently amplifies the likelihood of collisions among packets from neighboring nodes. Such collisions culminate in congestion at the Media Access Control (MAC) layer, which in turn substantially degrades transmission efficiency. Furthermore, when the buffer queue reaches capacity, nodes must discard incoming data packets, exacerbating the packet loss rate. This scenario not only underscores the challenges of efficiently managing network traffic but also emphasizes the critical need for sophisticated mechanisms to alleviate congestion and optimize packet handling, thereby ensuring network reliability and performance.

1. Load of Node

To measure the load situation of the current node, we propose the concept of *load of node* (LN), which is defined as follows:

(9) $$LN_{i}=\frac{Load_{i}(t)}{Load_{max}}$$

Among them, $Load_{i}(t)$ represents the length of data packet frames waiting to be processed in the MAC layer buffer queue of node i at time t, and $Load_{max}$ represents the maximum frame length that the MAC layer of node i can accommodate. It indicates that the larger the value of $LN_{i}$, the higher the utilization rate of the MAC layer buffer queue of node i at the current time, in other words, the greater the load on node i.

2. Load of Link

The load situation of a link is determined jointly by the load situation of the sender and receiver at both ends of the link. Therefore, in combination with load of node mentioned before, *load of link* (LL) is introduced to reflect the link load condition between two nodes, defined as follows:

(10) $$LL_{ij}=e^{LN_{i}}\cdot e^{LN_{j}}$$

In the above equation, $LL_{ij}$ represents the load of link between node i and node j, while $LN_{i}$ and $LN_{j}$ respectively represent the load of node i and node j. It reflects that the greater the load on the nodes at both ends of the link, the greater the load on the link.

3. Load of Neighbor Set

The load situation of a node is not only affected by the node itself and a certain link, but also by the neighbors around it. Therefore, we propose *load of neighbor set* (LNS) in order that reflecting the load impact of the neighbors around the node.

(11) $$LNS_{i}=\prod_{N_{i}}e^{LN_{i}}$$

Among them, $LN_{i}$ represents the load of neighboring nodes for node i, and $N_{i}$ represents the number of neighboring nodes for node i at time t.

### 3.2. Evaluation Algorithm of TOPSIS-MPR

Leveraging the valuable information extracted from received HELLO and TC packets, nodes within the network compute key evaluation metrics such as LD, SDL, ANSCR, LL, and LNS. In most cases, there is no neighbor node whose various indicators are superior to others, so it is necessary to weigh multiple indicators and select the best MPR nodes from the neighbor set.

- Construct the original evaluation matrix M.

With n neighbors of a node in the network as the evaluation objects, we select the LD, SDL, ANSCR, LL, and LNS of neighbors as evaluation metrics. The original evaluation matrix $M_{n\times 5}$ is constructed as follows:

(12) $$M_{n\times 5}={\left[d_{ij}\right]}_{n\times 5}={\left[\begin{array}{c} LD_{1}\\ LD_{2}\\ \vdots \\ LD_{n} \end{array}\begin{array}{c} SDL_{1}\\ SDL_{2}\\ \vdots \\ SDL_{n} \end{array}\begin{array}{c} ANSCR_{1}\\ ANSCR_{2}\\ \vdots \\ ANSCR_{n} \end{array}\begin{array}{c} LL_{1}\\ LL_{2}\\ \vdots \\ LL_{n} \end{array}\begin{array}{c} LNS_{1}\\ LNS_{2}\\ \vdots \\ LNS_{n} \end{array}\right]}_{n\times 5}$$

- Construct the standardized matrix N.

Since the dimensions and attribute types of each evaluation metric vary, direct comparison of the original data is not feasible. To calculate and compare various evaluation metrics, it is necessary to normalize the original evaluation matrix to obtain a standardized evaluation matrix.

Parameter types can be broadly categorized into benefit-type and cost-type. Benefit-type parameters have values that are better when they are larger, while cost-type parameters have values that are better when they are smaller. In the original evaluation matrix $M_{n\times 5}$, LD and SDL belong to the benefit type parameters, while ANSCR, LL, and LNS belong to the cost type parameters.

After normalization, the standardized evaluation matrix $N_{n\times 5}$ is obtained as follows:

(13) $$N_{n\times 5}={\left[nd_{ij}\right]}_{n\times 5}$$

(14) $$nd_{ij}=\left\{\begin{array}{l} \frac{d_{ij}-\underset{1\leq i\leq n}{\min}\{d_{ij}\}}{\underset{1\leq i\leq n}{\max}\{d_{ij}\}-\underset{1\leq i\leq n}{\min}\{d_{ij}\}},j\in \{LD,SDL\}\\ \frac{\underset{1\leq i\leq n}{\max}\{d_{ij}\}-d_{ij}}{\underset{1\leq i\leq n}{\max}\{d_{ij}\}-\underset{1\leq i\leq n}{\min}\{d_{ij}\}},j\in \{ANSCR,LL,LNS\} \end{array}\right.$$

- Construct the weight matrix W.

The entropy weighting method is employed to construct the weight matrix W. This is an objective weighting method that eliminates subjectivity and obtains high-precision weights. The core of this method is to associate the entropy value of evaluation indicators with the weight value. The more scattered the data, the greater the difference, and the smaller the entropy value, which means that the indicator carries more discriminative information and the weight of the indicator is greater.

The weight matrix $W_{1\times 5}$ constructed by the entropy weight method is as follows:

(15) $$\left\{\begin{array}{l} p_{ij}=\frac{nd_{ij}}{\sum_{i=1}^{n}nd_{ij}}\\ E_{j}=-\frac{1}{\ln(n)}\sum_{i=1}^{n}p_{ij}\ln(p_{ij}) \end{array}\right.,j=\{1,2,\cdots ,5\}$$

(16) $$W_{1\times 5}={\left[w_{j}\right]}_{1\times 5},w_{j}=\frac{1-E_{j}}{5-\sum_{k=1}^{5}E_{k}},j=\{1,2,\cdots ,5\}$$

- Construct the weighted evaluation matrix R.

By using the weight matrix $W_{1\times 5}$ and the standardized evaluation matrix $N_{n\times 5}$, we obtain the weighted evaluation matrix $R_{n\times 5}$ as follows:

(17) $$R_{n\times 5}={\left[r_{ij}\right]}_{n\times 5},r_{ij}=w_{j}\cdot nd_{ij},i=\left\{1,2,\cdots ,n\right\},j=\left\{1,2,\cdots ,5\right\}$$

- Determine the theoretical optimal solution $O^{+}$ and the worst-case solution $O^{-}$.

(18) $$\left\{\begin{array}{l} O_{j}^{+}=\underset{1\leq i\leq n}{max}r_{ij}\\ O_{j}^{-}=\underset{1\leq i\leq n}{min}r_{ij} \end{array}\right.,j=\{1,2,\cdots ,5\}$$

- Calculate the proximity factor matrix C.

Based on the Euclidean distance formula, we separately calculate the distance between each evaluation object and the theoretical optimal solution and the theoretical worst solution. The expressions are as follows:

(19) $$d_{i}^{+}=\sqrt{\sum_{j=1}^{5}{\left(r_{ij}-O_{j}^{+}\right)}^{2}}$$

(20) $$d_{i}^{-}=\sqrt{\sum_{j=1}^{5}{\left(r_{ij}-O_{j}^{-}\right)}^{2}}$$

Calculate the proximity factor c and obtain the proximity factor matrix $C_{n\times 1}$:

(21) $$C_{n\times 1}={\left[c_{i}\right]}_{n\times 1},c_{i}=\frac{d_{i}^{-}}{d_{i}^{+}+d_{i}^{-}},i=\left\{1,2,\cdots ,n\right\}$$

### 3.3. Specific Steps of TOPSIS-MPR Algorithm

N(A) represents the set of 1-hop neighbors of node A; $N_{2}(A)$ represents the set of 2-hop neighbors of node A; M(A) represents the MPR set of node A; $S_{2}(A)$ represents the judgment flag for M(A) to fully cover $N_{2}(A)$.

**Figure 5:**
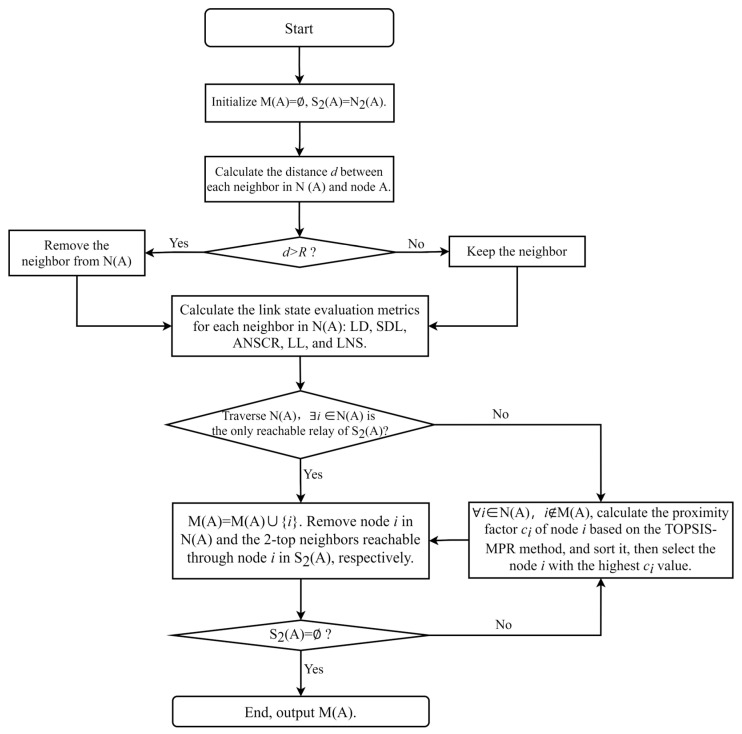
The flowchart of the TOPSIS-MPR algorithm.

As shown in Figure 5, this is the flowchart of the TOPSIS-MPR algorithm:

To facilitate understanding of the algorithm flowchart, the specific steps are described as follows:

**Step 1.** Initialize M(A)=∅, $S_{2}(A)=N_{2}(A)$.
**Step 2.** By traversing N(A), calculate the distance d between node A and its neighbors, and judge whether d is greater than the communication distance R. If so, remove the neighbor from N(A); otherwise, keep the neighbor.
**Step 3.** By traversing N(A), calculate link state evaluation indicators, namely: LD, SDL, ANSCR, LL, and LNS.
**Step 4.** ∃i∈N(A), so that node i is the only reachable relay of a node in $S_{2}(A)$, then add node i to M(A), that is: M(A)=M(A)∪{i}, and remove node i in N(A) and the 2-top neighbors in $S_{2}(A)$ reachable through node i, then proceed to the Step 6.
**Step 5.** ∀i∈N(A), i∉M(A), calculate the proximity factor $c_{i}$ of all nodes i based on the evaluation algorithm of TOPSIS-MPR, select the node i with the highest $c_{i}$ value, then add node i to M(A), that is: M(A)=M(A)∪{i}, and remove node i in N(A) and the 2-top neighbors in $S_{2}(A)$ reachable through node i, then proceed to the Step 6.
**Step 6.** Judge $S_{2}(A)=\emptyset$? If so, proceed to the Step 7; otherwise, proceed to the Step 5.
**Step 7.** The algorithm ends and M(A) is obtained, which is the MPR set of node A.

## 4. Simulation and Results

To validate the algorithm’s performance, the proposed CEL-OLSR protocol based on the TOPSIS-MPR algorithm is compared with PM-OLSR [32], ML-OLSR [33], LD-OLSR [18] and the standard OLSR protocol. Through simulation experiments, their differences in network performance metrics are analyzed, including packet delivery rate, average end-to-end delay, network throughput, and routing control overhead.

### 4.1. Simulation

Due to limitations such as the environment and interference, this paper conducts simulation experiments using a discrete event network simulator software called NS-3 running on a Linux environment. The simulated task scenario is range search or exploration missions performed by drones in UANETs. It involves setting up 50 mobile nodes in a 1.5 km × 1.5 km × 0.1 km area, with a three-dimensional Gaussian–Markov mobility model. The scenario tasks involve two sending nodes transmitting data packets of size 256 bytes to two other nodes at a const bit rate (CBR). The simulation experiment runs for 300 s.

The main simulation parameters are detailed in Table 1. To mitigate errors stemming from random factors in simulation experiments, each scenario undergoes five repetitions using distinct random number seeds, with subsequent averaging of results.

In the NS-3 simulator version 3.33, the ns3.33/scr/olsr/model module mainly consists of 7 files, including “olsr-header.h”, “olsr-repositories.h”, “olsr-routing-protocol.h”, “olsr-state.h”, “olsr-header.cpp”, “olsr-routing-protocol.cpp”, and “olsr-state.cpp” files.

**Figure 6:**
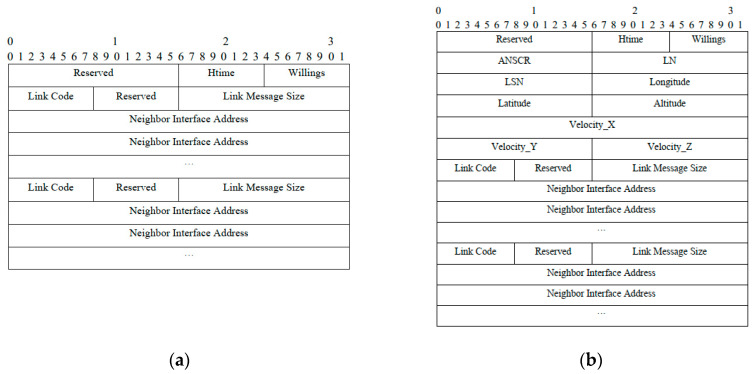
The Structure of hello message packet. (**a**) The structure of the original hello message package; (**b**) the structure of the improved and optimized hello message package.

In the network implementing the CEL-OLSR routing protocol, the interaction of node link state information among mobile nodes is achieved through hello control packets and TC control packets. In “olsr-header.h” and “olsr-header.cpp” files, we mainly modify the structure of the hello control message packet by adding the relevant evaluation parameters mentioned in Section 3 into the hello packet. In this simulation experiment, certain modifications are made to the structure of the hello message packets, as illustrated in Figure 6 below.

- ANSCR is the average neighbor set change rate of the sending node of the hello control packet. LN and LSN are the load of lode and the load of neighbor set of the node of the hello control packet, respectively.
- Longitude, Latitude, and Altitude are the position coordinates of the node in the x, y, and z directions of the sending node of the hello control packet, respectively.
- Velocity_X, Velocity_Y, and Velocity_Z are the velocities of the node in the x, y, and z directions of the sending node of the hello control packet, respectively.

In “olsr-repositories.h” file, it contains a series of tuples (including IfaceAssocTuple, NeighborTuple, TwoHopNeighborTuple, MprSelectorTuple, DuplicateTuple, TopologyTuple, AssociationTuple, and LinkTuple). We create a new tuple called LinkQosTuple, which is used to record link quality (including neighbor node main address, average neighbor set change degree ANSCR, link duration LD, stability degree of link SDL, load of node LN, and load of link LL).

In “olsr-state.h” and “olsr-state.cpp” files, they define the “OlsrState” class and various types of information tables (including LinkSet, NeighborSet, TwoHopNeighborSet, TopologySet, MprSet, MprSelectorSet, DuplicateSet, IfaceAssocSet, and AssociationSet). We create a new information table called LinkQosSet to store the link status of different nodes at different times. According to the five evaluation parameters proposed in Section 3, during the network simulation process, we directly store the relevant data in the LinkQosSet information table from the hello message packets exchanged between nodes or calculate and store them.

In “olsr-routing-protocol.h” and “olsr-routing-protocol.cpp” files, we calculate the link duration of node based on the speed and location information in the hello data packets, and compute the load of link between two nodes based on the load of node.

### 4.2. Analysis of Results

To evaluate the quality of the routing protocol design, this paper considers two aspects: data transmission accuracy and transmission speed. It selects the following four indicators to assess whether the optimized routing protocol can better meet the network performance requirements in the set task scenario.

1. Packet Delivery Rate

**Figure 7:**
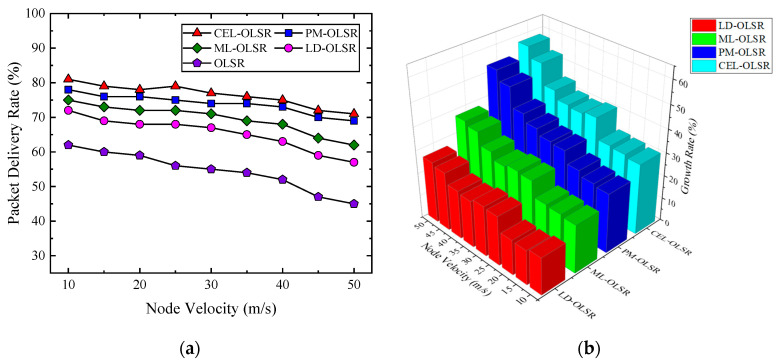
The comparison of packet delivery rate. (**a**) Trends in packet delivery rate for various protocols with changing speeds; (**b**) growth rate of packet delivery rate for various protocols compared to OLSR at different speeds.

Figure 7a illustrates the packet delivery rate for five protocols under varying speeds, while Figure 7b depicts the growth rate in packet delivery rate for CEL-OLSR, PM-OLSR, ML-OLSR, and LD-OLSR when compared to OLSR. Notably, CEL-OLSR demonstrates the highest packet delivery rate. When compared to PM-OLSR, CEL-OLSR exhibits an average increase in packet delivery rate of 2.56%. Compared to ML-OLSR, this increase is 6.89%. Furthermore, when compared to LD-OLSR and OLSR, CEL-OLSR registers an average improvement of 11.11% and 22.04%, respectively. In our simulation, we categorize the drone flight phase into three stages based on speed: low speed, mid-low speed, and mid-high speed. Table 2 presents the packet delivery rate for the comparison protocols at different speed stages. It is evident that the packet delivery rate decreases as speed increases. Importantly, CEL-OLSR exhibits the smallest decrease. The probability of link disruption rises with node speed, leading to an increase in the number of lost packets.

In the environment of ad hoc networks, the stability between links is a crucial factor in ensuring smooth packet delivery. With the increase in node mobility, the stability of links faces challenges because the relative positions of nodes become more variable, leading to continuous changes in network topology. This dynamic variation forces routing protocols to update frequently to adapt to the new network state, which may lead to the occurrence of routing loops, outdated routing information, and potential packet loss before reaching the destination. Additionally, as link disruption events increase, ensuring successful packet delivery becomes more difficult, often requiring multiple retransmissions. This not only exacerbates network load pressure but also, in cases of repeated unsuccessful retransmissions, may result in eventual packet discarding. In summary, the acceleration of node mobility inevitably exacerbates network dynamics, leading to a series of link and routing issues that may negatively impact successful packet delivery.

2. Average End-to-End Delay

**Figure 8:**
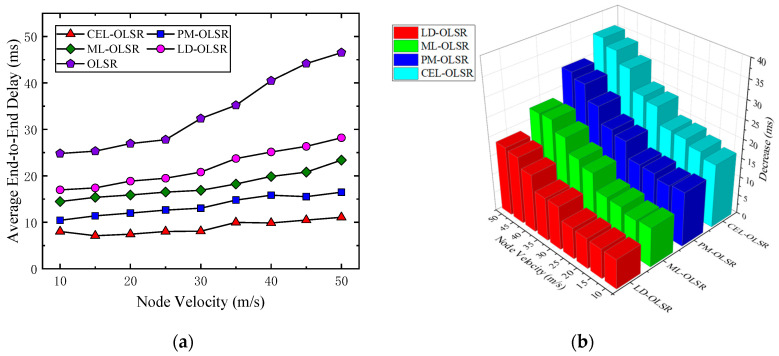
The comparison of average end-to-end delay. (**a**) Trends in average end-to-end delay for various protocols with changing speeds; (**b**) decrease amount of average end-to-end delay for various protocol compared to OLSR at different speeds.

As depicted in Figure 8a, within the node speed range of 10 m/s to 50 m/s, CEL-OLSR exhibits a maximum average end-to-end delay of approximately 11.07 ms, characterized by minimal fluctuations. In comparison to PM-OLSR, CEL-OLSR demonstrates a reduction in average end-to-end delay by 4.66 ms. Compared to ML-OLSR, this reduction amounts to 9.03 ms. Furthermore, when contrasted with LD-OLSR and the standard OLSR, CEL-OLSR showcases an overall decrease of 12.98 ms and 24.82 ms, respectively.

Figure 8b illustrates the reduction in average end-to-end delay achieved by CEL-OLSR, PM-OLSR, ML-OLSR, and LD-OLSR in comparison to OLSR.

As shown in Table 3, detailed results of end-to-end average delay for each protocol at different speed stages are presented. It is evident that transmission delay increases with rising speed, and CEL-OLSR exhibits a relatively gradual trend of delay variation.

When the node’s mobility rate increases, the network’s topology undergoes more frequent changes, leading to the breakage of established routing paths. This situation forces routing protocols to initiate a new round of route discovery processes, introducing additional time delays and thus prolonging the overall packet transmission time. In this dynamically changing network environment, data packets being transmitted may need to queue in relay nodes’ buffers to await updated routing information, which also contributes to increased delays. Furthermore, due to link instability, packet loss and subsequent necessary retransmissions further contribute to delays. In summary, the increase in node speed results in more frequent changes to the network topology, not only increasing the time required for route discovery and maintenance but also leading to delays from factors such as link rebuilding, buffer queuing, and packet retransmission, collectively raising the overall transmission latency of the network.

3. Throughput

**Figure 9:**
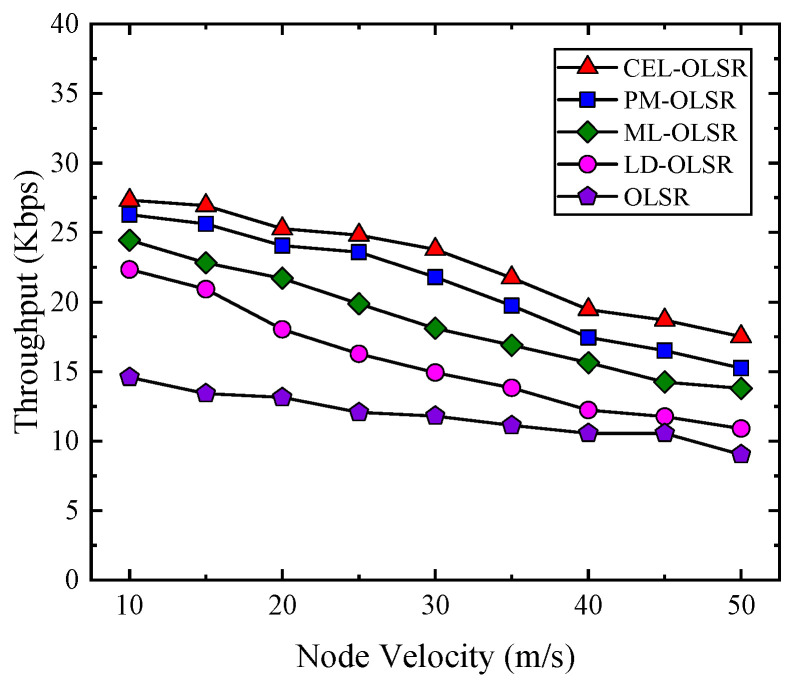
The comparison of network throughput among CEL-OLSR, PM-OLSR, ML-OLSR, LD-OLSR, and OLSR protocols.

As the node speed increases, the probability of link breakage and packet loss also both are increased, resulting in a significant decrease in throughput. As shown in Figure 9, compared with PM-OLSR, ML-OLSR, LD-OLSR, and the standard OLSR, the throughput of the CEL-OLSR protocol has increased by an average of 8.04%, 22.71%, 45.55%, and 93.19%, respectively. The results of throughput for comparison protocols at different speed stages are shown in Table 4.

The rapid movement of nodes contributes to heightened instability in network links, resulting in frequent disconnections and complicating sustained communication. Such dynamic motion exacerbates link disruptions, posing challenges to stable communication, and diminishing the efficiency of data transmission. Furthermore, the frequent link changes lead to packet loss, necessitating repeated retransmissions. This not only consumes bandwidth that could be allocated to new data packets but also increases time overhead. Within this dynamically evolving network environment, competition for wireless channels may intensify, elevating the likelihood of MAC layer collisions and further diminishing data transmission speeds, thereby reducing overall network throughput. In summary, heightened node mobility gives rise to a range of issues including link instability, frequent routing modifications, packet retransmissions, channel contention, congestion, and buffering delays, all of which significantly diminish network throughput.

4. Route Control Overhead

**Figure 10:**
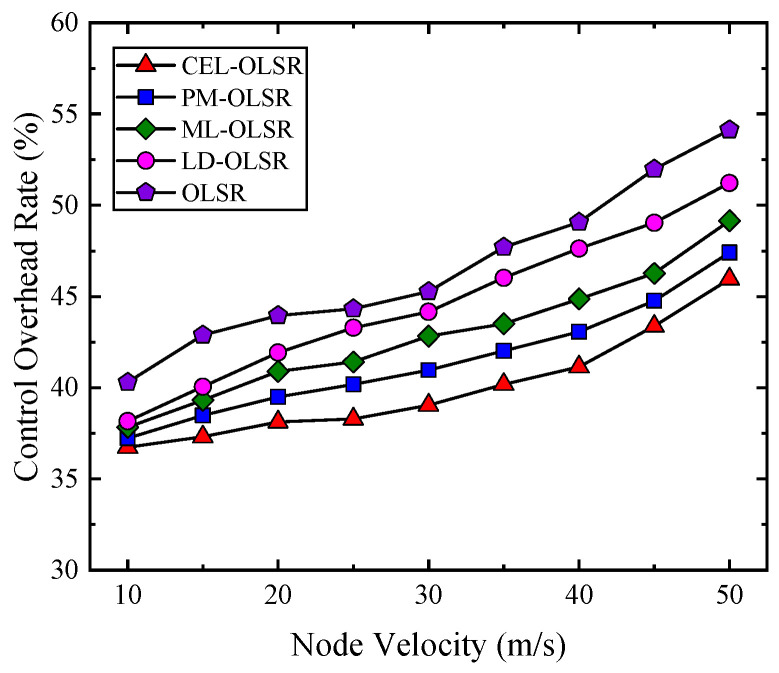
The comparison of control overhead rate among CEL-OLSR, PM-OLSR, ML-OLSR, LD-OLSR, and OLSR protocols.

The control overhead ratios for each protocol are depicted in Figure 10. Overall, the average cost ratios of control messages for CEL-OLSR, PM-OLSR, ML-OLSR, LD-OLSR, and OLSR protocols are 40.02%, 41.51%, 42.90%, 44.61%, and 41.16%, respectively. The detailed results for each protocol at different speed stages can be found in Table 5. A lower proportion of control messages implies a higher volume of actual data packet transmissions, which aligns with our expectations. While the overhead for CEL-OLSR also gradually increases with rising node speed, the rate of increase in control overhead slows down.

In high-speed mobile environments, frequent changes in network topology necessitate continuous updating and exchanging of routing information, resulting in a significant increase in control message overhead. In such environments, the intensification of link instability leads to more common occurrences of link breaks and reconstructions, compelling the system to transmit more link state control messages to maintain the accuracy of link information. Simultaneously, in the quest for optimal paths, the system must escalate the frequency of path detection and confirmation operations, further relying on the frequent exchange of control messages. The instability of links and path alterations also heighten the packet loss rate, prompting more retransmission requests, which often necessitate additional control messages for coordination. Additionally, the dynamically changing network environment demands more sophisticated congestion control and traffic management mechanisms to effectively administer network resources and circumvent congestion. Consequently, as node mobility increases, to uphold communication stability and efficiency, the system must augment the frequency of sending control messages, thereby significantly consuming network bandwidth and escalating node energy consumption. This surge in overhead is particularly pivotal in energy-constrained wireless network environments.

## 5. Conclusions

In UANETs, nodes often exhibit high mobility characteristics. Assuming node deployment remains relatively fixed, the emergence of “hotspot nodes” in the network due to mission requirements can lead to network congestion and a decline in network performance, among other issues. To mitigate these potential problems, we can address the situation by considering both node mobility and actual load conditions. By taking into account both mobility and load aspects, considering the influence of multiple factors, and making decisions based on comprehensive impact factors, we can enhance link stability, balance network load, and improve network performance.

Considering the importance of MPR nodes in OLSR, this paper takes a holistic approach from the perspective of mobility and load. It designs a comprehensive MPR evaluation algorithm based on link state awareness, selecting five evaluation metrics: Link Duration (LD), Stability Degree of Link (SDL), Average Neighbor Set Change Rate (ANSCR), Load of Link (LL), and Load of Neighbor Set (LNS). The weight coefficients are determined using the entropy weight method, and a comprehensive evaluation is conducted using the method of TOPSIS. Additionally, an optimized routing protocol named CEL-OLSR is proposed, which is aimed to solve the problem of single metric for MPR node selection in OLSR routing protocol. Compared to PM-OLSR, ML-OLSR, LD-OLSR, and OLSR, CEL-OLSR, respectively, improved packet delivery rates by an average of 2.56%, 6.89%, 11.11%, and 22.04%. Within the node speed range of 10 m/s to 50 m/s, CEL-OLSR exhibited the maximum average end-to-end delay of around 11.07 ms, with minimal fluctuation. In comparison to PM-OLSR, ML-OLSR, LD-OLSR, and OLSR, CEL-OLSR reduced the overall average end-to-end delay by 4.66 ms, 9.03 ms, 12.98 ms, and 24.82 ms, respectively. Furthermore, CEL-OLSR increased network throughput by 8.04%, 22.71%, 45.55%, and 93.19% compared to PM-OLSR, ML-OLSR, LD-OLSR, and OLSR, respectively. Overall, the average cost ratios of control messages for CEL-OLSR, PM-OLSR, ML-OLSR, LD-OLSR, and OLSR protocols are 40.02%, 41.51%, 42.90%, 44.61%, and 41.16%, respectively. As the node speed increases, the control overhead of CEL-OLSR also gradually increase. Overall, CEL-OLSR presents a considerable improvement in terms of delivery rates, latency, throughput, and routing efficiency over the compared protocols.

Route communication is a multifaceted challenge, wherein network communication quality and overall performance are shaped by link statuses, influenced by various factors. Concurrently, drones have the potential to function as mobile base stations or relay nodes, facilitating the establishment and extension of ground-based 5G networks. Consequently, by considering task-specific needs and the distinctive characteristics of drone ad hoc networks, optimizing the integration of drone networks with 5G technology can enhance data transmission efficiency, elevate packet delivery rates, minimize transmission delays, and boost network throughput. This trajectory is poised to emerge as a pivotal research avenue for UANETs in the foreseeable future.

## Figures and Tables

**Table 1:** The primary simulation parameters settings.

Simulation Parameter	Parameter Value
Simulation Time	300 s
Node Movement Range	1.5 km × 1.5 km × 0.1 km
Node Number	50 nodes
Mobility Model	3D Gaussian-Markov model
MAC Protocol	IEEE 802.11 b
Propagation Loss Model	Free space propagation loss model
Channel Rate	11 Mbps
Data Packet Size	256 bytes
Data Packet Rate	32,768 bps
Average Node Speed	10, 15, …, 50 m/s

**Table 2:** Results of packet delivery rate (PDR) for comparison protocols at different speed stages.

Speed Stage	Speed	CEL-OLSR	PM-OLSR	ML-OLSR	LD-OLSR	OLSR
Low speed stage: 10 m/s~20 m/s	10 m/s	81%	78%	75%	72%	62%
	15 m/s	79%	76%	73%	69%	60%
	20 m/s	78%	76%	72%	68%	59%
Mid low-speed stage: 25 m/s~35 m/s	25 m/s	79%	75%	72%	68%	56%
	30 m/s	77%	74%	71%	67%	55%
	35 m/s	76%	74%	69%	65%	54%
Mid high-speed stage: 40 m/s~50 m/s	40 m/s	75%	73%	68%	63%	52%
	45 m/s	72%	70%	64%	59%	47%
	50 m/s	71%	69%	62%	57%	45%

**Table 3:** Results of average end-to-end delay (AEED) for comparison protocols at different speed stages.

Speed Stage	Speed	CEL-OLSR	PM-OLSR	ML-OLSR	LD-OLSR	OLSR
Low speed stage: 10 m/s~20 m/s	10 m/s	8.0115 ms	10.4237 ms	14.4682 ms	16.9842 ms	24.8149 ms
	15 m/s	7.1125 ms	11.3785 ms	15.3781 ms	17.3932 ms	25.2993 ms
	20 m/s	7.4568 ms	11.9879 ms	15.8749 ms	18.8631 ms	26.9474 ms
Mid low-speed stage: 25 m/s~35 m/s	25 m/s	8.0299 ms	12.6382 ms	16.5078 ms	19.4923 ms	27.7827 ms
	30 m/s	8.0965 ms	13.0215 ms	16.8876 ms	20.8086 ms	32.3468 ms
	35 m/s	9.9706 ms	14.7824 ms	18.2349 ms	23.7232 ms	35.1833 ms
Mid high-speed stage: 40 m/s~50 m/s	40 m/s	9.8539 ms	15.8246 ms	19.8537 ms	25.1414 ms	40.4587 ms
	45 m/s	10.4783 ms	15.5259 ms	20.8123 ms	26.3367 ms	44.1619 ms
	50 m/s	11.0763 ms	16.4649 ms	23.3782 ms	28.1896 ms	46.5146 ms

**Table 4:** Results of throughput for comparison protocols at different speed stages.

Speed Stage	Speed	CEL-OLSR	PM-OLSR	ML-OLSR	LD-OLSR	OLSR
Low speed stage: 10 m/s~20 m/s	10 m/s	27.3271 Kbps	26.2906 Kbps	24.4521 Kbps	22.3386 Kbps	14.5856 Kbps
	15 m/s	26.9345 Kbps	25.6102 Kbps	22.8175 Kbps	20.9329 Kbps	13.4219 Kbps
	20 m/s	25.2784 Kbps	24.0471 Kbps	21.7056 Kbps	18.0408 Kbps	13.1396 Kbps
Mid low-speed stage: 25 m/s~35 m/s	25 m/s	24.8241 Kbps	23.5923 Kbps	19.8765 Kbps	16.2763 Kbps	12.0620 Kbps
	30 m/s	23.7894 Kbps	21.7894 Kbps	18.1109 Kbps	14.9236 Kbps	11.8063 Kbps
	35 m/s	21.7406 Kbps	19.7406 Kbps	16.9021 Kbps	13.8289 Kbps	11.1214 Kbps
Mid high-speed stage: 40 m/s~50 m/s	40 m/s	19.4539 Kbps	17.4539 Kbps	15.6454 Kbps	12.2358 Kbps	10.5553 Kbps
	45 m/s	18.7141 Kbps	16.5141 Kbps	14.2456 Kbps	11.7693 Kbps	10.5621 Kbps
	50 m/s	17.5242 Kbps	15.2542 Kbps	13.7857 Kbps	10.8997 Kbps	9.0364 Kbps

**Table 5:** Results of control overhead rate for comparison protocols at different speed stages.

Speed Stage	Speed	CEL-OLSR	PM-OLSR	ML-OLSR	LD-OLSR	OLSR
Low speed stage: 10 m/s~20 m/s	10 m/s	36.73%	37.23%	37.83%	38.17%	40.31%
	15 m/s	37.31%	38.48%	39.31%	40.05%	42.89%
	20 m/s	38.12%	39.49%	40.89%	41.93%	43.96%
Mid low-speed stage: 25 m/s~35 m/s	25 m/s	38.28%	40.19%	41.42%	43.28%	44.32%
	30 m/s	39.04%	40.97%	42.83%	44.16%	45.26%
	35 m/s	40.28%	42.01%	43.51%	46.02%	47.71%
Mid high-speed stage: 40 m/s~50 m/s	40 m/s	41.14%	43.07%	44.86%	47.62%	49.06%
	45 m/s	43.37%	44.76%	46.27%	49.04%	51.98%
	50 m/s	45.96%	47.41%	49.15%	51.22%	54.14%

## Data Availability

Data are contained within the article.
